# Multidisciplinary approaches to gliosarcoma: A case report and review of the literature

**DOI:** 10.1002/ccr3.5985

**Published:** 2022-08-23

**Authors:** Collin M. Labak, Nicholas M. Rabah, Jasmine P. Kipke, Uma V. Mahajan, Kyle B. Labak, S. Ahmed Ali, Nicole Fowler, Andrew E. Sloan

**Affiliations:** ^1^ Department of Neurosurgery University Hospitals Cleveland Medical Center, Case Western Reserve University Cleveland Ohio USA; ^2^ Case Western Reserve University School of Medicine Cleveland Ohio USA; ^3^ Bryn Mawr College Bryn Mawr Pennsylvania USA; ^4^ Department of Otolaryngology University Hospitals Cleveland Medical Center, Case Western Reserve University Cleveland Ohio USA; ^5^ Seidman Cancer Center and Case Comprehensive Cancer Center Cleveland Ohio USA

**Keywords:** extracranial, glioblastoma, gliosarcoma, multidisciplinary approach, reconstruction

## Abstract

A 58‐year‐old right‐handed man presented to our tertiary care center with gliosarcoma (GS) infiltration through the dura, skull, and soft tissue. Patient had a previous history of right temporal GS, with four intracranial surgeries prior to presentation. A multidisciplinary approach was used to treat the lesion and perform reconstruction.

## Graphical abstract

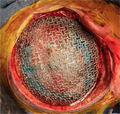

## 1 INTRODUCTION

Gliosarcomas (GS) are rare, aggressive primary brain tumors associated with extremely poor prognosis.^1^ Unlike most primary brain tumors, metastatic disease is the most frequent cause of death—even after resection and adjuvant treatment, recurrence is common.^2^, ^30^ They are defined as a type of glioblastoma (GBM), accounting for about 1.8%–2.8% of GBMs and 2%–8% of all malignant gliomas^3^, ^4^, ^5^ and are most common in men aged 40–70 years of age.^6^, ^7^ GS most frequently arises in frontotemporal brain parenchyma,^4^, ^5^, ^6^, ^7^, ^8^ and are composed of two cell populations: a glial cell population more akin to a typical GBM, and a spindle‐cell, sarcomatous component, with recent studies showing common genetic features between the cell populations.^4^, ^9^, ^10^, ^11^, ^12^

Intraoperatively, GS can be firm and well‐circumscribed, with the sarcomatous portion of the lesion at times resembling meningiomas, whereas the gliomatous component will have irregular, diffuse invasion into surrounding cerebral tissue, limiting complete resection.^5^, ^13^, ^14^ GS typically metastasizes via hematogenous spread,^7^, ^15^ although there are reported cases of spread via CSF to spinal cord or leptomeninges.^16^ The dura mater provides a natural barrier and gliosarcoma rarely extends beyond the dura into the cranium except in the case of prior radiation, craniotomy, or invasive procedure.^1^, ^2^, ^12^, ^17^, ^18^ Because extracranial metastasis is uncommon, understanding of the factors affecting spread and how to prevent it is limited.^1^ Here, we present a patient with GS infiltration through the dura, skull, and overlying soft tissue and dermis in order to discuss the importance of a multidisciplinary approach to treating these lesions, as well as possible hypotheses for this uncommon direct extension into calvarium and scalp.

## 2 CASE REPORT

The patient is a 58‐year‐old right‐handed man without significant past medical history who presented to our Midwest tertiary care center with previous history of right temporal gliosarcoma who had a total of four intracranial surgeries prior to his presentation to our clinic. Initially, he presented to an outside hospital with seizures. That hospital performed index resection in June 2020, and he underwent adjuvant radiation and temozolomide at the same institution. His index resection was at the outside hospital in June 2020, and he underwent adjuvant radiation and temozolomide at the same institution. He had two further resections following this surgery. The second surgery was in October 2020; it was complicated by wound infection requiring washout at which time native bone was left in place. The patient also was found to have bony metastasis in the cervical spine for which he underwent corpectomy and instrumentation in January 2021 prior to a third debulking in February 2021.

**FIGURE 1:**
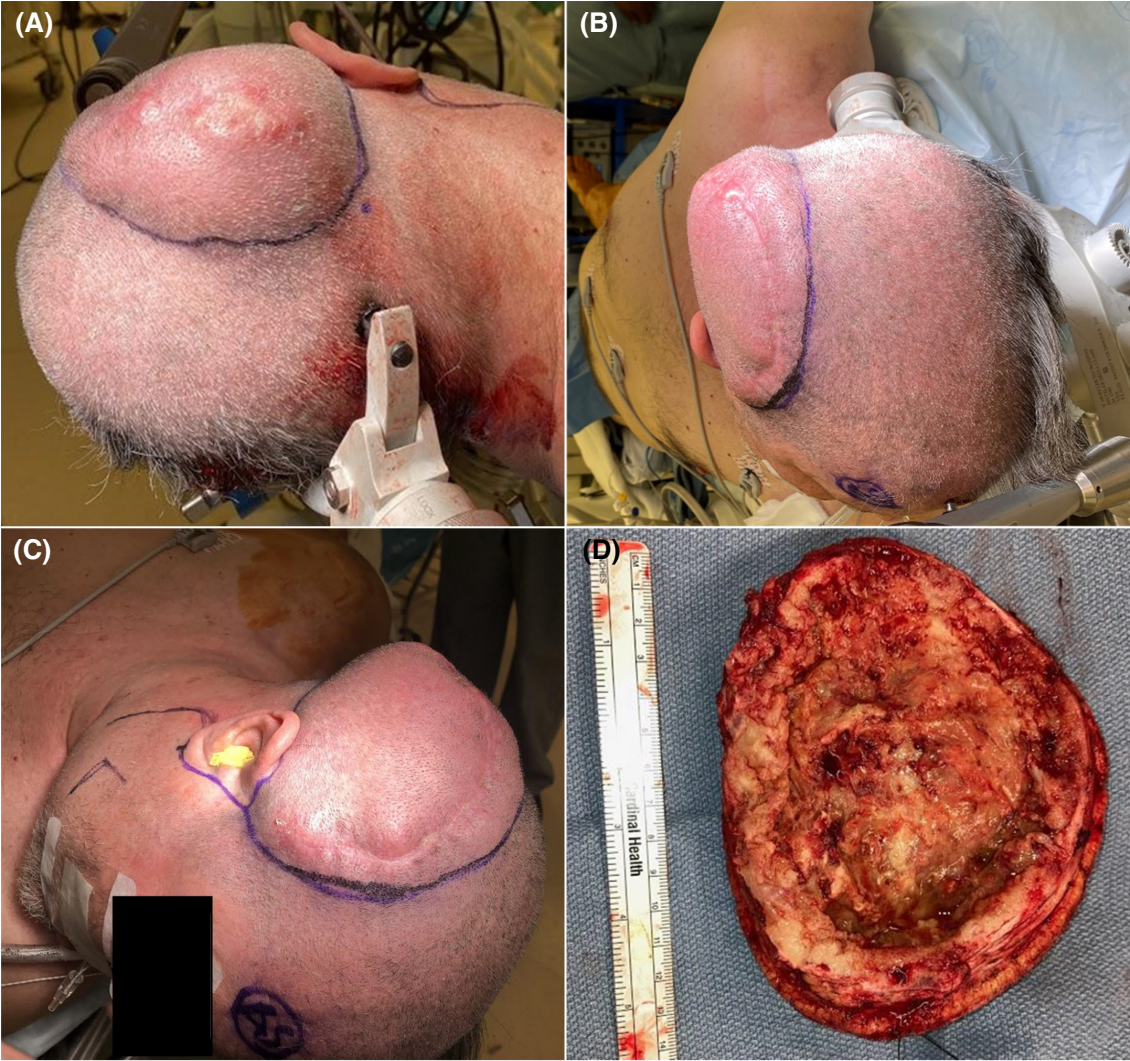
Preoperative view of soft tissue invasion of tumor and previous craniotomy scar (A‐C). Internal surface of excised scalp with substantial tumoral invasion (D)

The patient was initially evaluated by video telecommunication by our team in April 2021; his chief complaint during his virtual visit was scalp firmness and pain. He was admitted to our center shortly thereafter for in‐person evaluation and surgical planning. The otolaryngology/head and neck surgical service evaluated the patient in‐house for planning. Magnetic resonance imaging (MRI) and venogram (MRV) were obtained. Compared to previous imaging, there was substantial tumor recurrence within the resection cavity and peri‐resection bed invasion, as well as enhancement within dura and surrounding bone including petrous temporal, parietal, and occipital bones, and soft tissue of the scalp, which correlates to the gross findings seen on physical examination (Figure 1A–C).

**FIGURE 2:**
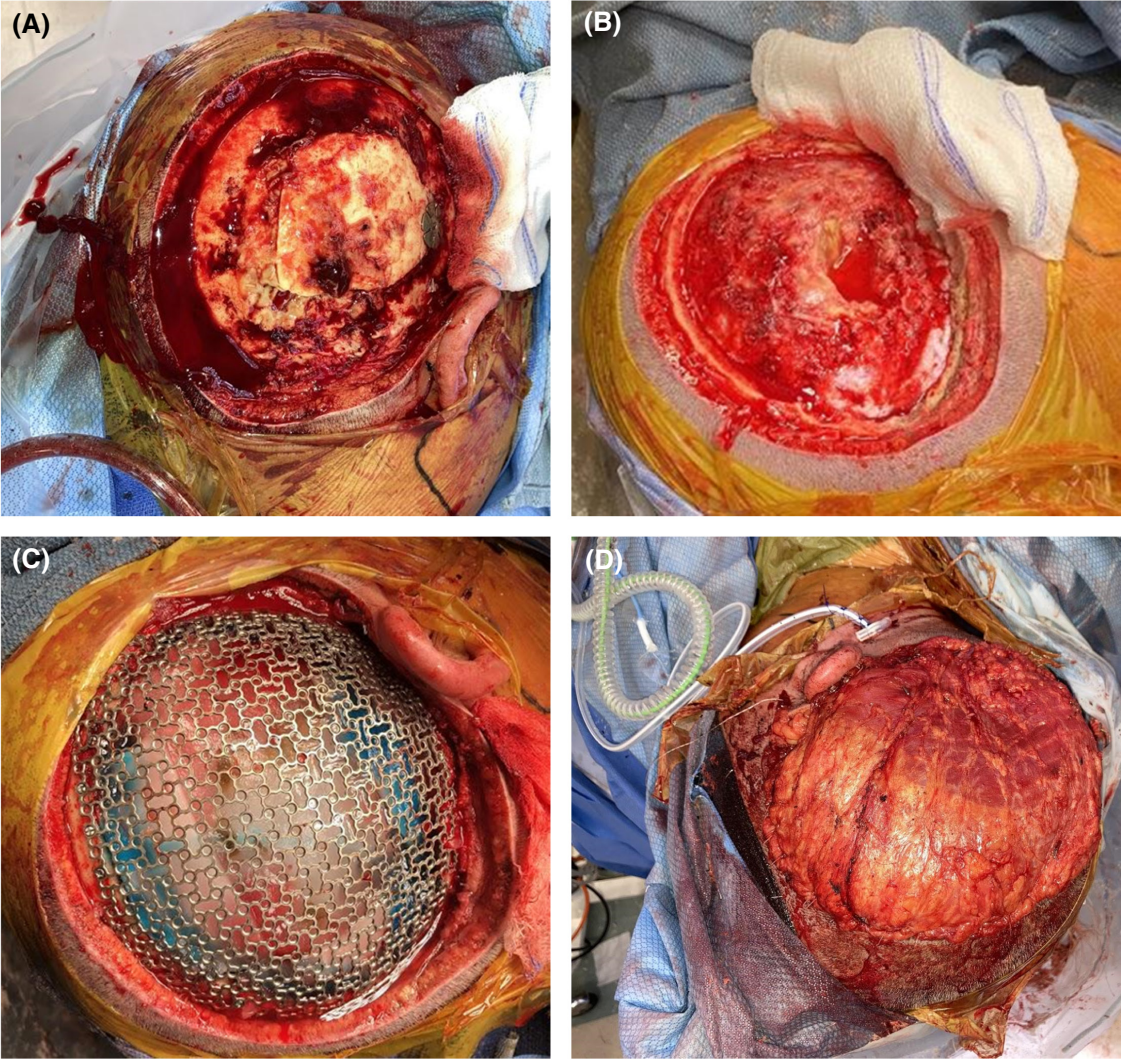
Previous craniotomy site with fixation hardware in place (A). Expanded craniectomy to include all bone underlying scalp excision (B). Titanium mesh cranioplasty placed over resection site (C). Latissimus dorsi free flap within the site of excision (D)

Two days after admission, after appropriate imaging was obtained, the patient was taken for operative management. Management was multidisciplinary, with different specialties collaborating on operative treatment. First, the head and neck team outlined and performed a wide local excision on a 14‐cm section of scalp that grossly appeared to have tumoral invasion (Figure 1A–D). The superficial temporal artery and its paired vein were identified and ligated with vessel clips. Then, the previous cranial fixation hardware was removed from patient's previous surgical site (Figure 2A), and the bone flap was removed. The craniectomy was further expanded to include all grossly dysplastic bone (Figure 2B). The petrous temporal bone was also drilled out, and pneumatized bone was sealed with bone wax. After bony removal, an extensive removal of invaded dura mater and intracranial tumor debulking was carried out, with grossly abnormal tissue removed. Further resection with 5‐aminolevulinic acid was completed. Following this resection, dural onlay was used to cover resection bed, and a titanium mesh cranioplasty was placed over craniectomy defect and fixated to native bone (Figure 1C). Then, the head and neck surgery team proceeded to harvest a latissimus dorsi free flap with anastomosis from thoracodorsal pedicle to superficial temporal recipient. This flap was tacked into place and bolstered with Xeroform (Figure 2D).

Postoperatively, a lumbar drain was placed for 72 h of CSF diversion for flap protection as well as CSF leak prophylaxis, as the temporal bone was significantly resected. The patient had an unremarkable postoperative course and was discharged to skilled nursing facility. Final pathology showed gliosarcoma invasion in scalp, dura, and bone. Scalp resection was extensively sampled with evidence that radial margins were free of tumor.

## 3 DISCUSSION

Given the rarity of gliosarcomas with respect to central nervous system tumors, much of the literature describing their diagnosis, treatment, and management is limited to case reports and small retrospective studies. The case description above demonstrates a unique presentation of gliosarcoma—one that involves extracranial presentation by way of direct extension through the dura mater, skull, and soft tissues, distant metastases, as well as a treatment strategy for addressing the extracranial extension.

Currently, the World Health Organization classification system includes gliosarcoma as a subtype of GBM. Limited literature suggests, however, that gliosarcoma exhibits features, which may warrant clinical distinction including histologic and radiologic appearance, as well as its propensity to metastasize. Case reports and small series have reported metastases to the lung, liver, cervical lymph nodes, spleen, adrenal glands, bone marrow, kidneys, oral mucosa, skin, skull, ribs, and spine.^19^, ^20^, ^21^, ^22^, ^23^, ^24^, ^25^, ^26^, ^27^, ^28^, ^29^, ^30^ Specifically, in contrast to GBM, the sarcomatous portion of gliosarcoma is hypothesized to hematogenously spread to extracranial sites.^30^ In the present case, it is likely that distant metastasis to the CS vertebral body was likely hematogenous in nature as well. Two possible scenarios, as described by Yokoyama et al.^29^ might account for this particular patient's postoperative metastases: (1) tumoral spread through the venous system prior to surgery through direct extension or introduction into the venous system during surgery, (2) direct seeding of the dura mater, skull and scalp during surgery with subsequent invasion into vasculature. Much like the case presented by Yokoyama et al.^29^ with massive extension into the skull and soft tissues abutting the operative site, the second option is most plausible. Interestingly, this patient did have extensive invasion of his tumor into the right transverse sinus, with complete obliteration of the venous drainage on the side of his tumor.

As a direct consequence of the lack of treatment and outcome data for GS, and its current classification as a glioblastoma subtype, the treatment of gliosarcoma exactly mirrors that of GBM. In the present case, that treatment paradigm was followed upon initial presentation to the outside hospital, including an attempt at gross total resection followed by adjuvant radiation and temozolomide. However, when combining this case's ultimate metastatic presentation at our institution, similar case series describing metastatic presentations after index surgeries, and the known propensity of the sarcomatous portion to metastasize, attempt at surgical resection may have to be weighed against the risk of metastasis. This thought process may be expounded upon when considering the recent development of targeted molecular therapeutics in the treatment cancer, including PIK3 inhibitors (alpelisib) and PTEN inhibitor, two genes found to have a high prevalence of mutation in sequenced tumor samples.^31^ In conclusion, it is important to consider a multidisciplinary approach when treating highly invasive tumors such as GS, and to be mindful of mechanisms of spread while planning resection in index surgeries.

## AUTHOR CONTRIBUTIONS

Collin M. Labak, M.D participated in surgery, assisted with drafting paper. Nicholas M. Rabah, Jasmine P. Kipke, Uma V. Mahajan, and Kyle B. Labak assisted with drafting and revisions. S. Ahmed Ali, M.D, Nicole Fowler, M.D, and Andrew E. Sloan, M.D performed surgery, reviewed manuscript.

## CONFLICT OF INTEREST

The authors have no conflicts of interest to report at this time.

## CONSENT

Written informed consent was obtained from the patient to publish this report, in accordance with the journal's patient consent policy.

## Data Availability

Data sharing not applicable – no new data generated.
